# Cave‐Dwelling Planariidae in Dinaric Karst Exhibit Differing Levels of Cave Trait Evolution

**DOI:** 10.1002/ece3.74488

**Published:** 2026-10-28

**Authors:** Lucija Kauf, Miquel Vila‐Farré, Fruszina Ficze‐Schmidt, Emilija Bakula, Jochen C. Rink, Helena Bilandžija

**Affiliations:** ^1^ Ruđer Bošković Insititute Zagreb Croatia; ^2^ Department of Tissue Dynamics and Regeneration Max Planck Institute for Multidisciplinary Sciences Göttingen Germany; ^3^ Faculty of Biology and Psychology Georg‐August‐University Göttingen Göttingen Germany

**Keywords:** cave fauna, Dinarides, planaria, troglomorphic adaptation, variability

## Abstract

The Dinaric karst encompasses a network of over 10,000 caves and represents one of the world's most important subterranean biodiversity hotspots. It is inhabited by remarkably diverse and often endemic species, including planarian flatworms. Although cave planarians have long been known from the region, they have not yet been investigated using an integrative approach, and their evolutionary relationships with surface‐dwelling relatives remain unresolved. To address these gaps, we combined field sampling, phylogenetic analyses based on the *COI* and *18S* genes, and phenotypic characterisation. Our results show that cave planariids in the Dinaric karst belong to at least three genera and are more widespread and diverse than previously assumed. We increased the number of cave records in the Dinaric karst from 26 to 37 and documented cf. *Atrioplanaria* and *Phagocata* in Croatian caves for the first time. Phylogenetic reconstructions are consistent with independent cave colonisation events, including multiple instances within the genera *Crenobia* and cf. *Atrioplanaria*. Variation in pigmentation and eye reduction, both within and between populations, further reveals heterogeneous evolutionary trajectories of cave‐associated phenotypes. Our molecular phylogeny and documentation of trait variability constitute a first step towards establishing Planariidae as a model for investigating the mechanisms underlying the convergent evolution of pigment loss and eye reduction in cave environments.

## Graphical abstract

The Dinaric karst of Croatia is a global hotspot of subterranean biodiversity, yet its cave planarians remain poorly studied. By combining field sampling, phylogenetic analyses of mitochondrial *COI* and nuclear *18S* markers, and analyses of pigmentation and eye reduction, we provide the first integrative assessment of cave planariids in Croatia and reveal substantially greater diversity than previously recognised, including evidence for multiple independent cave colonisation events. Pronounced variation in cave‐associated traits within and between populations, together with complex biogeographical patterns, identifies planariids as a promising model for studying convergent evolution in subterranean environments.

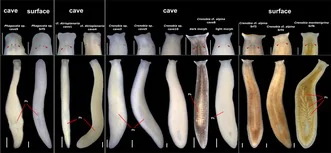

## 1 Introduction

Caves and subterranean ecosystems are found around the world, but because access is more restricted than in surface ecosystems, they remain among the least‐researched habitats. The Dinaric karst, a limestone formation running parallel to the eastern coast of the Adriatic Sea (Zupan Hajna 2019), is one of the world's best‐studied subterranean regions and one of the richest hotspots of subterranean biodiversity (Culver and Sket 2000; Culver et al. 2006; Deharveng et al. 2009; Gibert and Deharveng 2002; Năpăruș‐Aljančič et al. 2026; Pipan et al. 2020). Nevertheless, many taxonomic groups remain poorly investigated, and new species and lineages continue to be discovered. Due to its high biodiversity both above and below ground, the Dinaric karst provides a natural laboratory for investigating evolutionary processes such as speciation, radiation, phenotypic plasticity and convergence (Bedek et al. 2019; Bilandžija et al. 2013; Borko et al. 2021; Čupić et al. 2023; Hlebec et al. 2023; Lukić et al. 2020; Recknagel et al. 2024). With respect to the latter process, while cave‐dwelling organisms belong to many different phyla, their adaptations to cave environments tend to converge on a shared set of troglomorphic phenotypes (Protas and Jeffrey 2012), the most iconic of which are the loss of body pigmentation and visual structures. These are often accompanied by non‐visual sensory enhancements, changes in metabolism and neural and behavioural alterations (Protas and Jeffrey 2012). Mechanisms underlying the convergent evolution of cave‐associated traits have been elucidated in only a few examples, with a primary focus on pigment loss (Bilandžija et al. 2012, 2026; Protas et al. 2006, 2011).

Given their presence in both subterranean and surface realms of the Dinaric karst (de Beauchamp 1937; Gourbault 1972; Kenk 1936a, 1936b; Knezović et al. 2015; Komárek 1919, 1920; Mrázek 1904, 1907; Stanković 1926; Stanković and Komárek 1927), and amenability to mechanistic studies of both pigment biosynthesis and eye formation (He et al. 2017; Lindsay‐Mosher and Pearson 2019; Saad et al. 2025; Stubenhaus et al. 2016; Wang et al. 2016), freshwater planarians represent an ideal model for investigating the convergent evolution of cave‐related traits. Planarians are generally easy to maintain in laboratory cultures (Cebrià and Newmark 2005), with the high regenerative ability of many species (Reddien and Sánchez Alvarado 2025; Vila‐Farré et al. 2023) often offering an effective means of culture expansion. Since cave planarians share these traits with their surface counterparts, they are particularly suitable for functional and mechanistic studies. However, such studies require resolution of the phylogenetic relationships between surface and cave populations, with the identification of closely related cave and surface species as the ideal starting point for comparative analyses.

Even though research on planarian species in the Dinaric karst dates back to the beginning of the 20th century (Mrázek 1904, 1907), it has remained largely sporadic and non‐systematic (Gourbault 1972; Komárek 1919, 1920; Stanković 1926; Stanković and Komárek 1927), with only a few exceptions (Knezović et al. 2015; Vila‐Farré et al. 2026). So far, 26 subterranean localities with Planariidae have been reported (Appendix S1) (Gourbault 1972; Kenk 1936b; Komárek 1919, 1920; Mrázek 1907; Sluys 2022; Stanković 1926; Stanković and Komárek 1927), comprising 22 caves and four springs. Altogether, nine different species and one variety, as well as one unidentified planariid, were recorded in these localities: *Atrioplanaria opisthogona* (Kenk, 1936), *Atrioplanaria racovitzai* (Beauchamp, 1928), *Crenobia anophthalma* (Mrazek, 1907), *Crenobia montenigrina* (Mrazek, 1904), *Phagocata bosniaca* (Stanković, 1926), *Phagocata dalmatica* (Stanković & Komárek, 1927), *Phagocata illyrica* (Komárek, 1919), *Phagocata ochridana* (Stanković & Komárek, 1927), *Planaria torva* (Müller, 1773) and *Planaria torva* var. *stygia* (Kenk, 1936) (Gourbault 1972; Kenk 1936b; Komárek 1919, 1920; Mrázek 1907; Sluys 2022; Stanković 1926; Stanković and Komárek 1927). Only *Ph. dalmatica* and *C. anophthalma* were recorded within the territory of Croatia (Stanković and Komárek 1927; Vila‐Farré et al. 2026), while one record of *C. montenigrina* from the cave Golubnjača on Ujilica Mountain (Komárek 1919, 1920) lacks sufficient information to determine whether it is located in Croatia or Bosnia and Herzegovina (BiH).

Here we report the first molecular analysis of the biodiversity of cave planariids in the Dinaric karst, combining field sampling, phylogenetic reconstructions using mitochondrial *cytochrome oxidase subunit I* (*COI*) and nuclear *18S* genes, and characterisation of hallmark traits associated with life in caves (namely, reduction or loss of pigmentation and eyes). Resulting molecular phylogenies and patterns of trait variability within and between populations are consistent with multiple instances of cave colonisation by planariids in the Dinarides and heterogeneous evolutionary trajectories of cave‐related traits. Our work reveals greater planarian biodiversity than previously recognised in the Dinaric karst. It sets the stage for future comparative studies using cave Planariidae as a model for understanding mechanisms of convergent evolution.

## 2 Methods

### 2.1 Sample Collection

The cave specimens analysed in this study were collected by our team members and colleagues from the Croatian Biospeleological Society at 11 localities during extensive cave fauna surveys conducted over the past three decades. Our samples come from a portion of the Dinaric karst that encompasses mostly Croatia and includes two samples from BiH. However, because of the lack of group‐level studies in the literature, we discuss our findings in the context of the broader Dinaric karst. All sampling was authorised by national authorities of Croatia and BiH (Permit numbers 517‐05‐1‐1‐20‐05, 517‐10‐1‐1‐22‐5 and 07/1/625‐466/17). To minimise the impact on protected subterranean populations, the number of individuals that could be collected from each locality was limited by the sampling permits. Consequently, sample sizes were necessarily small (Appendix S2). Additionally, six surface locations were sampled for this study (Figure 1A,B, Appendix S2). Planarians were sampled using brushes, transfer pipettes and/or large pipettes (Vila‐Farré et al. 2026). Native water was collected when possible and used to gradually acclimate animals to a standardised formulation of planarian water (PW, Montjuïc recipe) (Cebrià and Newmark 2005) or cave planarian water (cPW, 1:4‐diluted PW in RO water) (Vila‐Farré et al. 2023). All cave animals were maintained in laboratory incubators without light at a constant temperature of 8°C or 12°C and fed monthly with *Asellus aquaticus* (Linnaeus, 1758). Surface animals were kept in the same conditions but fed calf liver. Depending on the population, animals were maintained in the laboratory for a few weeks to a few years.

**FIGURE 1:**
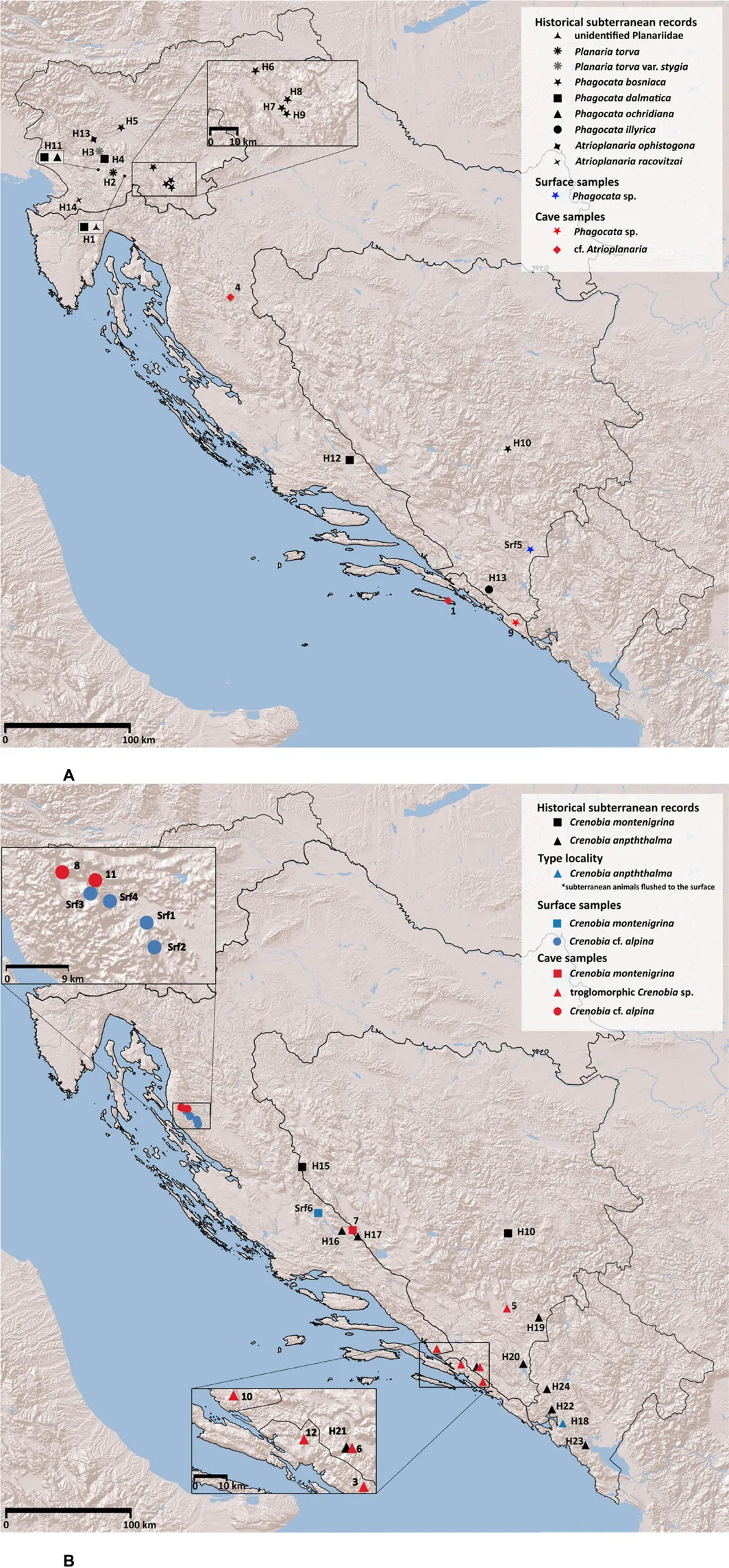
Map of historical and newly sampled localities of (A) *Phagocata* and cf. *Atrioplanaria* and (B) *Crenobia* in the Dinaric karst.

### 2.2 Imaging

Animals were imaged using a Canon EOS 6D Mark II camera equipped with a Canon EF 100 mm f/2.8L lens, or a Canon EDS 250D camera equipped with a Sigma 150 mm 1:2.8 APO Macro DG HMS lens for whole animal imaging of individuals larger than 2 mm. Images of smaller individuals, as well as detailed images, were taken using either a ZEISS Stereo Microscope Stemi 508 paired with a ZEISS Axiocam 208 colour digital camera or a Canon EDS 250D camera mounted on a Motic SZM‐171 stereo microscope.

### 2.3 DNA Extraction and PCR

Animals were starved for 3 weeks or more before DNA extraction and were either stored in 96–100% ethanol or processed directly. DNA extractions were performed on representative samples from each locality, following published protocols (Vila‐Farré et al. 2026) or using commercial DNA extraction kits (DNeasy Blood & Tissue Kit, Cat. No. 69504, Qiagen, Germany; Quick‐DNA Microprep Kit, Cat. No. D3020, Zymo Research, USA).

*COI* PCR amplification was performed using the published MVCOI900 primer pair and amplification protocol (Vila‐Farré et al. 2026), or with a *touch‐down* PCR amplification. *Touch‐down* PCR was conducted using commercial Taq MasterMixes (ALLin HS Red Taq Mastermix, Cat. No. HSM0301, highQu, Germany; GoTaq G2 Colorless Master Mix, Cat. No. M7832, Promega, USA). The reaction conditions were: (1) 5 min at 95°C, (2) 30 s at 95°C, (3) 30 s at 60°C, (4) 1 min at 70°C; (5) 30 s at 95°C, (6) 30 s at 46°C, (7) 1 min at 70°C and (8) 3 min at 70°C. Steps 2–4 were repeated for seven cycles, with the (3) temperature decreasing by 2°C per cycle; steps 5–7 were repeated for 30 cycles.

*18S* sequences were obtained by PCR amplification using NZYTaq II 2× Green Master Mix (Cat. No. MB35803, NZYTech, Portugal) with 18S_F1 and 18S_R9 primers (Álvarez‐Presas et al. 2008; Carranza et al. 1996). The reaction conditions were as follows: (1) 5 min at 95°C, (2) 30 s at 95°C, (3) 30 s at 45°C, (4) 2 min at 68°C and (5) 5 min at 68°C. Steps 2–4 were repeated for 30 cycles. All commercial kits were used according to the manufacturers' instructions.

Amplification products were analysed by gel electrophoresis on a 1% agarose gel and purified using the QIAquick PCR Purification Kit (Cat. No. 28104, Qiagen, Germany). The purified PCR products were Sanger‐sequenced by Macrogen using the amplification primers in both directions, with two additional internal primers (18S_4F and 18S_5R; Carranza et al. 1996) for *18S* due to the amplicon size. Low‐quality bases at the beginning and the end of the raw sequences were trimmed before assembly in Geneious Prime (version 2026.0.2) (Kearse et al. 2012). The final lengths of assembled *COI* sequences ranged from 367 bps to 905 bps, while the final lengths of assembled *18S* sequences ranged from 1632 bps to 1714 bps (Appendix S3). Predicted protein sequences were analysed to ensure the absence of internal stop codons in the *COI* gene, and all sequences were subsequently used in BLASTN (Altschul et al. 1990) queries against the NCBI database (Sayers et al. 2025).

### 2.4 Extraction of Sequences from Transcriptomes

Our dataset was complemented with additional Planariidae sequences from Planmine (Brandl et al. 2016; Rozanski et al. 2019), as well as from unpublished transcriptomes of cave planarians generated by our group (Appendix S2) via a BLAST search integrated in Geneious Prime (Kearse et al. 2012). The hits were manually inspected, and regions homologous to the *COI* and *18S* amplicons were extracted. Wherever both DNA‐amplified and transcriptome‐retrieved sequences were available for the same population, the transcriptome sequences were used. The length of the extracted *COI* sequences was 923 bps, while the length of the extracted *18S* sequences ranged from 1796 to 1809 bps (Appendix S3).

### 2.5 *COI* and *18S* NCBI Search

To complement our dataset, the NCBI nucleotide database (Sayers et al. 2025) was searched for *COI* and *18S* on 15.01.2026. The search was limited to the genera *Crenobia*, *Phagocata* and cf. *Atrioplanaria* based on the preliminary NCBI BLAST (Altschul et al. 1990) results and the transcriptome‐based phylogenetic trees in (Vila‐Farré et al. 2023, 2026). Additionally, sequences from the planariid *Polycelis felina* (Dalyell, 1814) were used as an outgroup because of its phylogenetic position (Vila‐Farré et al. 2023, 2026). Only the samples with both *COI* and *18S* were retained for subsequent analyses, and they originated from the work of Brändle et al. (2017) and Carranza et al. (1998, 1999).

### 2.6 Sequence Alignment and Phylogenetic Tree Reconstruction

In total, 72 sequences were analysed (16 generated in this study, seven extracted from published transcriptomes, and 49 retrieved from NCBI). Sequences of individual genes were aligned using MAFFT (v7.490) (Katoh and Standley 2013) (Algorithm G‐INS‐I; Scoring matrix 200PAM/k = 2; Gap open penalty = 1.53; Offset values = 0.123) integrated in Geneious Prime (Kearse et al. 2012). For the *COI* gene, the alignment was 1047 bps long, containing 39.20% missing data, 0.22% ambiguities and 0.04% gaps. The *18S* alignment was 1823 bp long, containing 46.66% missing data, 0.03% ambiguities and 1.06% gaps. The missing information in the alignment ends was substituted with Ns, and the sequences were concatenated into a single alignment using the integrated concatenation option in Geneious Prime (Kearse et al. 2012), generating a new alignment with a length of 2870 bps and a total of 43.98% missing data, 0.10% ambiguities and 0.69% gaps, further referred to as the ‘full’ alignment. To assess the effect of missing data on phylogenetic relationships, we constructed two additional trimmed alignments using an approach similar to that of Vila‐Farré et al. (2026). Positions in the ‘gap50’ alignment have an occupancy of 50% or more, with a length of 1092 bps and 5.39% missing data, 0.19% ambiguities and 0.03% gaps. The ‘trimmed’ alignment was obtained manually by trimming the alignment ends to include a region in which all sequences contained information. The final length of the ‘trimmed’ alignment was 782 bps, with 2.19% gaps and 0.16% ambiguities (no missing data). The reading frame for the *COI* gene was preserved in both alignments. Maximum Likelihood (ML) phylogenies (Yang and De Angelis 2013) were inferred from all three alignments using IQ‐TREE (version 3.0.1) (Nguyen et al. 2015). Alignment partitions were specified for *COI* codon positions 1, 2 and 3, and for the *18S* gene. Model selection was restricted to models supported by MrBayes, followed by tree construction using the parameters ‘‐m MFP ‐mset mrbayes’. Branch support was assessed with 10,000 ultrafast bootstrap replicates and an approximate Likelihood‐Ratio Test with 10,000 replicates (‘‐bnni ‐bb 10,000 ‐Alrt 10,000’). The trees were visually inspected using iTOL (version 7.5) (Letunic and Bork 2024). The same pipeline was used for phylogenetic analyses of each gene individually, with an appropriately adjusted partition file for *COI*, and exclusion of the partition file for the *18S* gene.

Based on ML results, we performed Bayesian inference (BI) on ‘full’ alignment only, since both ‘gap50’ and ‘trimmed’ showed a lack of resolution or relationships inconsistent with transcriptome‐based phylogenetic relationships from Vila‐Farré et al. (2023, 2026). Single‐gene trees were also run independently for the full‐length *COI* and *18S*. As determined by IQ‐TREE (Nguyen et al. 2015), the best models compatible with MrBayes for the *COI* + *18S* alignment were: GTR + F + G4 for *COI* codon position 1, HKY + F + G4 for *COI* codon position 2, GTR + F + I + G4 for *COI* codon position 3, and HKY + F + I + G4 for the *18S* partition. MrBayes (version 3.2.7) (Ronquist et al. 2012, 2019) was run in two independent runs with four chains for each of the alignments. The number of generations was 10,000,000, with a burn‐in of 10,000, and samples were taken every 1000 generations. Chain convergence was assessed first by observing the average standard deviation of split frequencies (< 0.01 for all alignments), as well as inspecting trace plots in Tracer (version v1.7.2) (Rambaut et al. 2018). The effective sample size (ESS) for all model parameters exceeded 625, as confirmed using the convenience package (version 1.0.0) (Fabreti and Höhna 2022) in R (version 4.4.3) (R Core Team 2021). Trees were created using the ‘‐allcompat’ function in MrBayes (Ronquist et al. 2012, 2019).

## 3 Results

### 3.1 Phylogenetic Relationships

The freshwater Tricladida samples collected from 11 cave localities (eight in Croatia and three in BiH), and six surface localities (five in Croatia and one in BiH) were analysed using genetic information on the *COI* and *18S* genes, external habitus and the number of pharynges.

To further characterise cave planariid diversity, we conducted BI and ML analyses on individual *COI* and *18S* datasets, as well as on concatenated *COI + 18S* alignments. Analyses based on individual genes, trimmed datasets and all ML reconstructions either lacked resolution or recovered relationships inconsistent with previously published transcriptome‐based phylogenies (Vila‐Farré et al. 2023, 2026; Appendices S4–S14). Therefore, subsequent interpretations were based on the BI analysis of the concatenated *COI + 18S* dataset (Figure 2).

**FIGURE 2:**
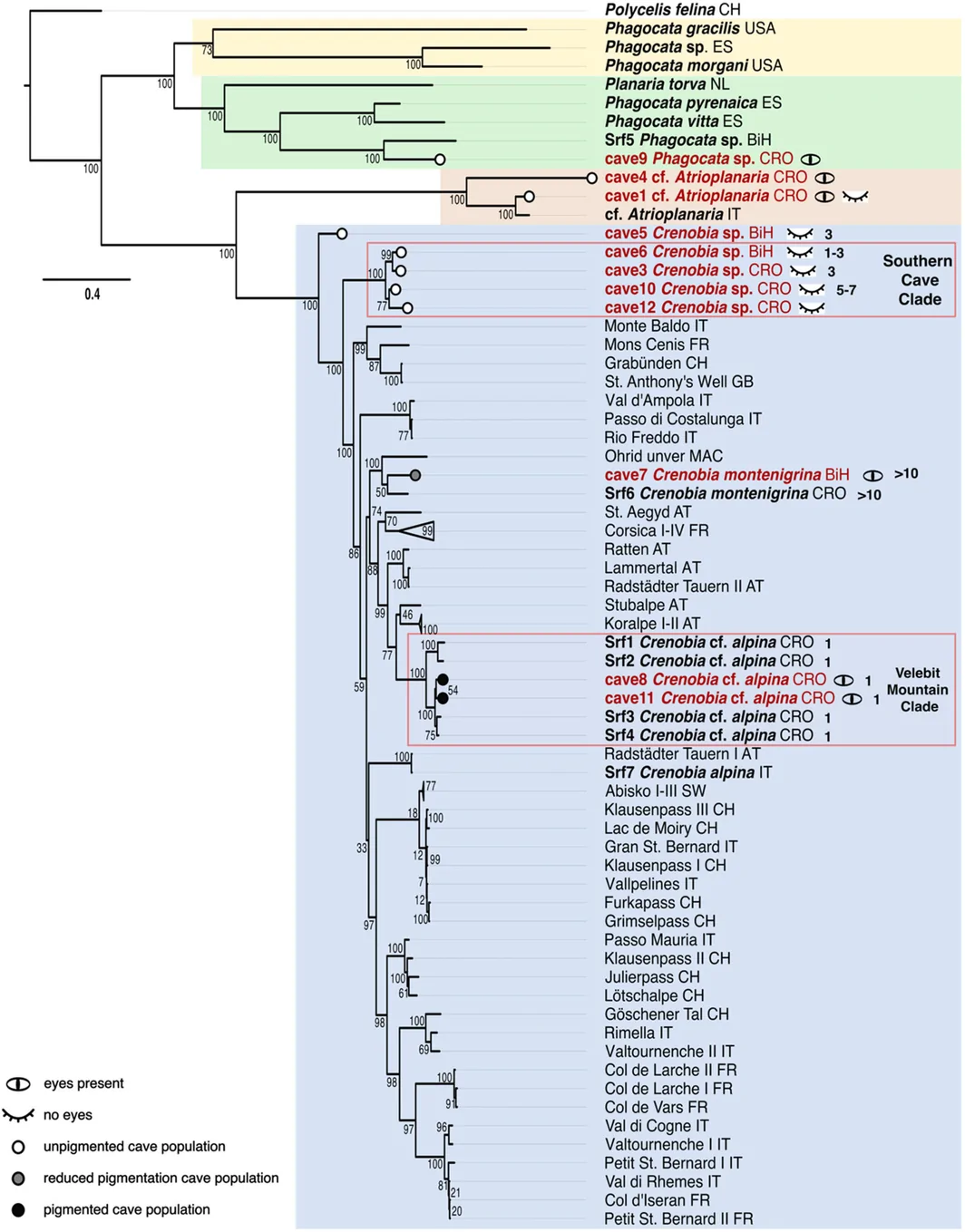
BI phylogenetic tree from *COI* and *18S* genes, using the ‘full’ alignment dataset. The values represent posterior probability. Genus *Crenobia* is shaded in blue and cf. *Atrioplanaria* in orange. European *Phagocata* and *Planaria torva* in green; American *Phagocata*, with a misassigned sample from Spain, in yellow. Cave populations are marked by red letters. Where available, the number of pharynges is indicated to the right of species names. The presence or absence of eyes was plotted in cave populations only.

This analysis placed the cave specimens in three lineages: cf. *Atrioplanaria*, *Crenobia* and a clade comprising *Phagocata* and *Planaria*. Previous transcriptome‐based phylogenetic studies reported the clustering of *Planaria torva* with the European species *Phagocata pyrenaica* (Vila‐Farré et al. 2023, 2026). Consistently, *P. torva* is basal to a cluster containing the European *Phagocata pyrenaica* , *Phagocata vitta* and specimens from cave9 and Srf5, with strong statistical support (green in Figure 2). The only European *Phagocata* outside this clade (*Phagocata* sp. ES; *COI* GenBank accession DQ666053, *18S* accession AF013150) suffers from a likely erroneous taxonomic assignment (Vila‐Farré et al. 2026); therefore, its placement among the American *Phagocata* should be interpreted cautiously. In summary, considering the clustering of cave9 and Srf5 with European *Phagocata* with strong support in all phylogenetic reconstructions (Figure 2, Appendices S4–S14), and their external morphology, we assign them to the genus *Phagocata* (Figure 3).

**FIGURE 3:**
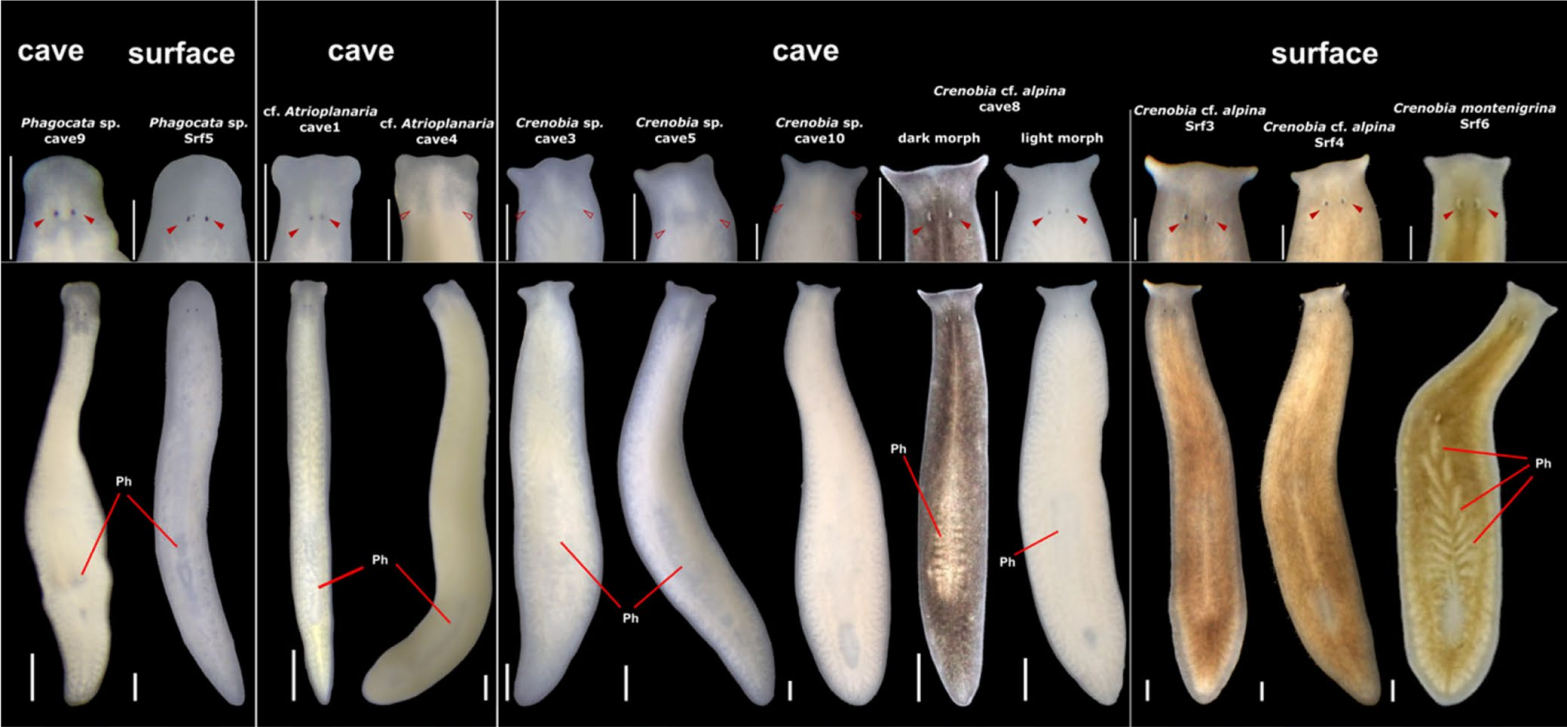
Live specimens of planariids collected during the expeditions. Top panel, anterior region (head) in close‐up; bottom panel, whole‐body habitus. Ph: pharynx/pharynges. Full arrowheads point to the visible eye spot. Empty arrowheads point to the area where the eye spot is expected but is not visible. Scale bar, 500 μm.

Specimens from cave1 and cave4 clustered with a previously described surface population from Sardinia (Vila‐Farré et al. 2023, 2026), and were therefore assigned to cf. *Atrioplanaria*. Their sister clade was *Crenobia*, which included specimens from eight caves and five surface localities. Relationships within *Crenobia* lineages remained only partially resolved due to low statistical support for several nodes. Nevertheless, cave specimens formed four distinct and supported lineages: (1) cave5, which occupied a sister position to all remaining *Crenobia*; (2) the Southern Cave clade, comprising specimens from cave3, cave6, cave10 and cave12; (3) cave7, which clustered with *Crenobia montenigrina*; and (4) cave8 and cave11, which grouped with nearby surface populations from Velebit Mountain (Figure 2).

### 3.2 Pigmentation and Eye Variation

The more prominent characteristics of cave‐adapted animals are the lack of body pigmentation and eyes. Analysis of this variation in our samples shows that both cave and surface *Phagocata* were unpigmented but possessed well‐developed eyes, consistent with previous reports of unpigmented epigean *Phagocata* (Dugès 1830; Vila‐Farré et al. 2011) (Figure 3). The two cf. *Atrioplanaria* populations were also unpigmented. Animals from cave4 were eyeless, while individuals from cave1 showed variability in eye number, ranging from zero to two eyes (Appendix S15). Within cave‐dwelling *Crenobia*, the two early‐branching groups (cave5 and the Southern Cave clade) lacked both body pigmentation and discernible eyes (Figure 3). In contrast, *C. montenigrina* from cave7 and both cave populations from the Velebit Mountain clade retained well‐developed eyes, but the Velebit Mountain clade showed varying levels of body pigmentation (Figure 4).

**FIGURE 4:**
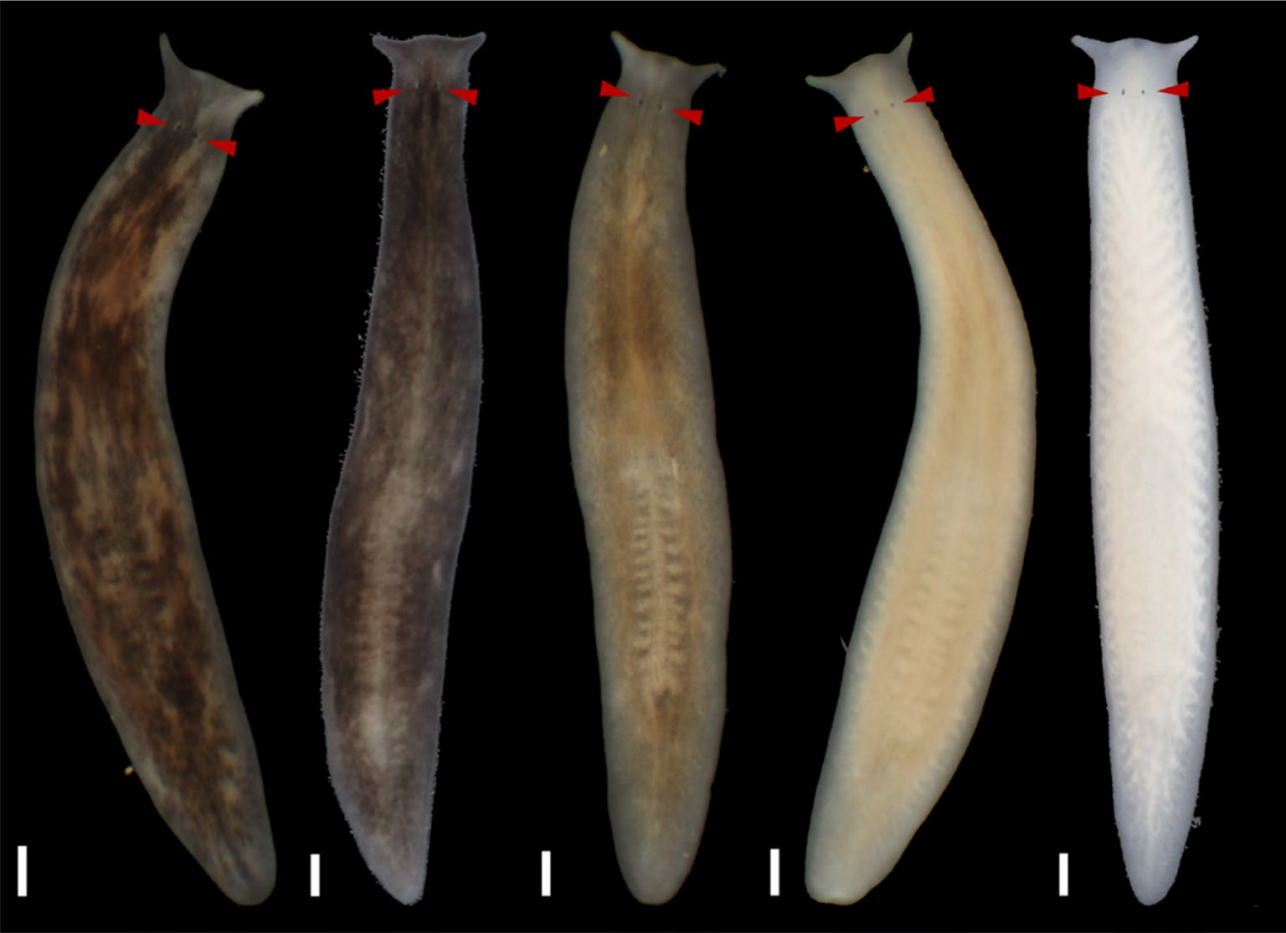
Intrapopulation variation in body pigmentation among living cave8 animals imaged under identical illumination conditions, with the increase in pigmentation levels from right to left. The eyes of the animals are marked with arrowheads. Scale bar, 500 μm.

### 3.3 Variation in the Number of Pharynges in *Crenobia*

To further characterise *Crenobia* diversity, we examined the number of pharynges, given the established taxonomic value of this trait (Sluys 2022). All animals from the Velebit Mountain clade were monopharyngeal (both cave and surface individuals), consistent with the description of *C. alpina* , but also *C. bathycola* (Steinmann, 1911) and *C. corsica* (Arndt, 1922). The remaining specimens were polypharyngeal. Animals from cave7 possessed more than 10 pharynges, consistent with previous descriptions of *C. montenigrina* (Chichkoff 1903; Mrázek 1904; Sluys 2022; Stella 1957). Animals from cave5 and the Southern Cave clade externally resembled *C. anophthalma*, the only known obligatory cave *Crenobia* species (Mrázek 1907; Sluys 2022), in their complete absence of pigmentation and eyes. However, they showed variability in the number of pharynges. While specimens from cave5, cave3 and cave6 possessed up to three pharynges, animals from cave10 had between five and seven pharynges (Figures 2 and 5; number of analysed animals in Appendix S2). Animals from cave12 were not examined due to insufficient material.

**FIGURE 5:**
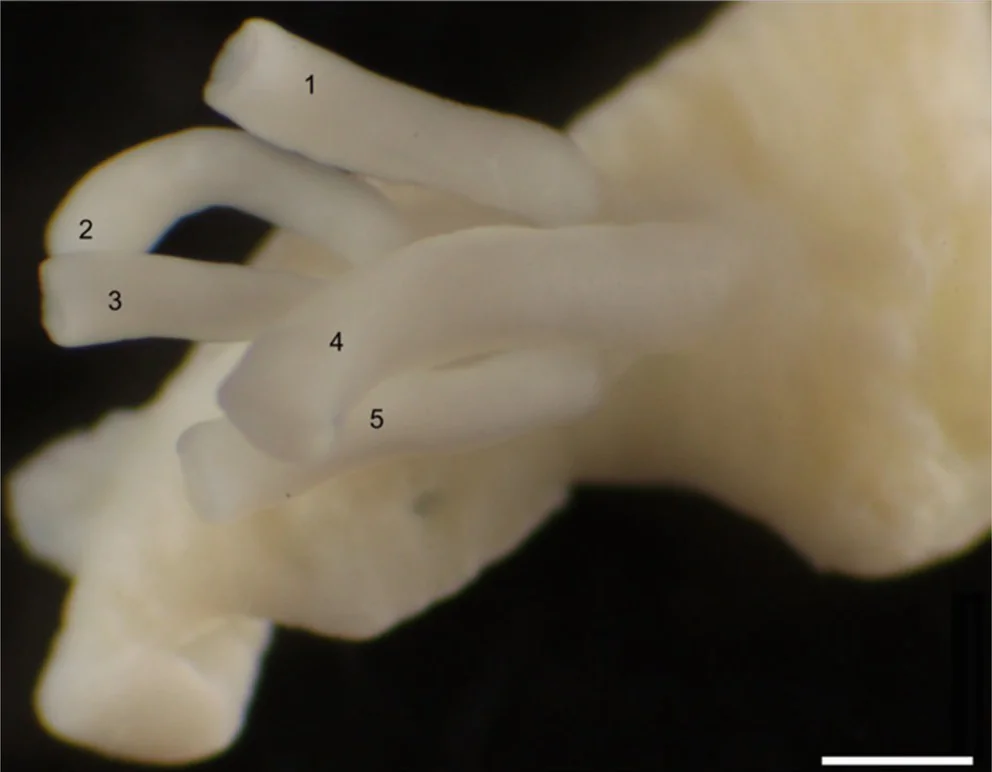
Expelled pharynges from an individual collected in cave10 after fixation in 96% ethanol. Most animals had five pharynges, with some having six or seven. Scale bar: 500 μm.

## 4 Discussion

Our sampling efforts revealed at least seven potentially independent Planariidae lineages in the caves of the Dinaric karst: two cf. *Atrioplanaria*, one *Phagocata* and four *Crenobia* (Figure 2). We provide the first cave record of *Phagocata* in Croatia, as the previously reported subterranean record of *Ph. dalmatica* in Croatia was collected in a spring (Stanković and Komárek 1927). Similarly, we provide the first evidence of the genus cf. *Atrioplanaria* in Croatian caves, while *C*. cf. *alpina* is documented for the first time in the Dinaride caves. These findings substantially increase the known subterranean diversity of planariids in Croatia, from a single species ( *C. anophthalma* ) and two cave localities to three genera across 11 caves, while the number of cave occurrences of Planariidae in the Dinaric karst rises to 37 localities. This notable increase underscores the species richness of the planarian fauna in Croatia (Vila‐Farré et al. 2026) and in the Dinaric karst.

Our phylogenetic analysis combined the rapidly evolving mitochondrial *COI* gene with the more conserved nuclear *18S* gene. Despite the low support of some branches, the placement of the main genera—cf. *Atrioplanaria*, *Crenobia*, *Phagocata* and *Planaria*—was broadly consistent with previous transcriptome‐based phylogenies (Vila‐Farré et al. 2023, 2026). Resolving relationships among clades within each genus, particularly within *Crenobia*, will require additional multigene or phylogenomic analyses combined with traditional taxonomic approaches. Nevertheless, our results suggest possible biogeographical patterns. In particular, the intriguing phylogenetic proximity between cf. *Atrioplanaria* from the island of Mljet (cave1; one of the southernmost Croatian islands) and a Sardinia population (Vila‐Farré et al. 2023), rather than specimens from the geographically closer locality in Croatia (cave4 population), is supported in all analyses (Figure 2, Appendices S4–S8, S10–S14), except for the ML inference on the ‘trimmed’ *COI* alignment (Appendix S9). Given the substantial distance between the two caves, and their association with distinct river catchment systems (the Adriatic and the Danube, respectively), a single cave colonisation event followed by subterranean dispersal seems unlikely. Instead, the Sardinian and Mljet (cave1) populations may represent descendants of a lineage formerly distributed across a wider area, ranging from the Eastern Adriatic to Sardinia, different from the one that originated the population in cave4. Under this scenario, cave1 and cave4 populations would represent independent cave colonisation events. Similar biogeographic and phylogenetic patterns have been reported in cave spiders (*Troglohyphantes*): *T. cyrnaeus* Isaia, 2023, from Corsica, forms a clade with lineages from the northern Dinaric karst (Isaia et al. 2023). Although the planarian populations examined occur in distinct microregions of the Adriatic and Sardo‐Corsican block, the observed pattern may reflect paleogeographic connections between the Dinaric karst and the western Mediterranean during the geological history of the Mediterranean Basin (Isaia et al. 2023).

Relationships within *Crenobia* remained largely unresolved in our analysis and will require additional molecular markers. Nevertheless, we recovered the clades presented in Brändle et al. (2017). Importantly, we identified four cave‐associated *Crenobia* lineages. The specimen from cave7 was assigned to *C. montenigrina* and was the only cave representative of this species included in the analysis. *Crenobia montenigrina* is a surface‐dwelling species that has rarely been reported from caves (Komárek 1919, 1920). If this taxonomic assignment is confirmed by further analyses, the occurrence of *C. montenigrina* as well as of the cave‐adapted *C. anophthalma* in the region (Gourbault 1972; Komárek 1919, 1920; Mrázek 1907; Sluys 2022) would support at least two independent cave‐colonisation events within the genus. Furthermore, the clustering of cave and surface populations within the Velebit Mountain clade as well as in the clade containing cave7, together with the phylogenetic position of cave5 specimens relative to other lineages, adds additional support to the hypothesis that *Crenobia* has colonised caves multiple times (Figure 2, Appendices S4, S5, S7–S10, S12 and S14).

Variation in eye number (*Atrioplanaria* and *Crenobia*) and pigmentation (*Crenobia*) warrants further investigation to clarify the evolution of these traits. In *Phagocata* and cf. *Atrioplanaria*, the absence of pigmentation cannot be interpreted as a result of cave adaptation, due to the wide occurrence of unpigmented forms in surface environments (Dugès 1830; Vila‐Farré et al. 2011, 2023). Variation in eye number between and within cf. *Atrioplanaria* cave populations (Figure 3, Appendix S15) suggests either ongoing or heterogeneous evolutionary processes. Similar to observations in cave1, intrapopulation eye‐phenotype variability has recently been documented in *Girardia multidiverticulata* de Souza, Morais, Cordeiro & Leal‐Zanchet, 2015 (Saad et al. 2025; de Souza et al. 2015). Interpopulation variability (cave1 vs. cave4) may reflect different stages of adaptation to cave environments, as eye loss has been associated with the time elapsed since cave colonisation (Recknagel and Trontelj 2022), and eyeless cave *Atrioplanaria* have previously been documented, for example, in Slovenia (Gourbault 1972). However, the relationship between trait loss and time since colonisation may be non‐linear (Borko et al. 2021; Delić et al. 2016; Leijs et al. 2012; Trontelj et al. 2012; Wessel et al. 2007). The observed variation could instead reflect location‐specific environmental pressures, or phenotypic plasticity in these populations. Although these possibilities cannot be distinguished with the available data, the variation documented here provides a basis for future studies examining how environmental conditions and evolutionary history shape trait reduction in cave planarians.

Trait variability in pigmentation and eye number is remarkable in *Crenobia*. Fully troglomorphic lineages (cave5 and Southern Cave clade) represent the most advanced degree of trait reduction in this genus. In contrast, *C. montenigrina* from cave7 shows partial reduction of body pigmentation (pale colour with a pink hue). Interestingly, the historical record of *C. montenigrina* from the cave Golubnjača (Ujilica Mountain) references animals that are ‘snow‐white, with degenerate eyes’ (Komárek 1920). These differences in trait evolution within a single species (*Crenobia montenigrina*) may reflect independent cave colonisation events or divergent selection pressures in different caves. Finally, the most extensive intrapopulation variability in pigmentation among cave‐dwelling *Crenobia* was recorded at Velebit Mountain (two populations; cave8 shown in Figure 4). Since the usual functions of body pigmentation, such as protection against solar radiation, camouflage or intraspecific communication, are ineffective in the absence of light, the observed high pigmentation variability might be a result of relaxed selective pressures (Wilkens 2023) and reflect a recent cave colonisation in Velebit Mountain. Alternatively, this variability could reflect ongoing gene flow from surface populations (Protas and Jeffrey 2012). However, since no planarians were found in a nearby surface stream leading to Ponor Štirovača (cave8), despite repeated sampling efforts, this explanation is unlikely.

Our results raise taxonomic questions within *Crenobia*. Sluys (2022) recently confirmed the species status of *C. alpina* , *C. corsica*, *C. bathycola*, *C. montenigrina* and *C. anophthalma*. To date, only *C. anophthalma* and *C. montenigrina* (one population) have been reported with certainty from Dinaric subterranean environments (Komárek 1920; Sluys 2022). However, our phylogenetic analyses indicates substantially greater diversity. Individuals from the Velebit Mountain clade (four surface and two cave populations) are likely related to a predominantly epigean species, *C. alpina* . To our knowledge, these specimens represent the first record of a monopharyngeal cave *Crenobia* in the Dinaric karst. In contrast, specimens from cave5 and most populations in the Southern Cave clade (cave3, cave6 and cave12) are similar to *C. anophthalma* concerning pigmentation, eye absence and pharynx number (Mrázek 1907, 1914; Sluys 2022), except for cave10 individuals, that have five to seven pharynges (Southern Cave clade). The broad morphological concordance, the occurrence of the two clades within or close to the known range of the species (Komárek 1919, 1920; Sluys 2022), and the existence of a historical *C. anophthalma* record at < 600 m from cave6 (Komárek 1919, 1920) suggest that these lineages could represent *C. anophthalma* populations. If cave10 individuals belong to *C. anophthalma* , the number of pharynges may be taxonomically less informative than previously assumed. The phylogenetic position of cave5 specimens, the sister clade to all other lineages, and the higher number of pharynges in cave10 individuals support the existence of additional subterranean species of *Crenobia* in the area. In either case, our findings highlight the need for an integrative taxonomic re‐evaluation of *Crenobia*, with the emphasis on cave populations (Sluys 2022; Vila‐Farré et al. 2026).

## 5 Conclusions

Our study demonstrates that cave planariids are more widespread and diverse in the Dinaric karst than previously known. The emerging phylogenetic and biogeographical patterns point to a complex evolutionary history and highlight the need for further research in the region, including broader taxonomic and geographic sampling. The newly generated gene sequences, particularly for *Crenobia*, provide a valuable reference for future DNA barcoding, phylogenetic and integrative taxonomic studies. Analyses incorporating additional molecular markers, together with detailed taxonomic studies, will be essential to resolve relationships within Planariidae and to delimit species boundaries. Finally, the evidence provided for multiple cave‐colonisation events and the emerging patterns of cave colonisation and variation in cave‐associated traits highlight the potential of Planariidae as a promising comparative system for investigating the evolutionary processes underlying convergent evolution in subterranean environments.

## Author Contributions

**Lucija Kauf:** conceptualization (equal), data curation (lead), formal analysis (lead), investigation (lead), visualization (lead), writing – original draft (lead), writing – review and editing (equal). **Miquel Vila‐Farré:** conceptualization (equal), investigation (equal), supervision (equal), validation (equal), writing – original draft (supporting), writing – review and editing (equal). **Fruszina Ficze‐Schmidt:** investigation (supporting), writing – review and editing (supporting). **Emilija Bakula:** investigation (supporting), writing – review and editing (supporting). **Jochen C. Rink:** funding acquisition (equal), project administration (equal), resources (equal), supervision (equal), writing – review and editing (equal). **Helena Bilandžija:** conceptualization (lead), funding acquisition (equal), project administration (equal), resources (equal), supervision (equal), writing – original draft (supporting), writing – review and editing (equal).

## Funding

This project was funded by the Max Planck Society (J.C.R.) and by the Tenure Track Pilot Programme of the Croatian Science Foundation, Ecole Polytechnique Fédérale de Lausanne and Project TTP‐2018‐07‐9675 Evolution in the Dark, with funds from the Croatian‐Swiss Research Programme (H.B.).

## Conflicts of Interest

The authors declare no conflicts of interest.

## Supporting information


**Appendix S1:** Historical records of the subterranean Planariidae in the Dinaric karst.

**Appendix S2:** List of localities and samples used in the phylogenetic analyses.

**Appendix S3:** List of samples collected for this study, with their respective sequence accession numbers, and additional information on the *COI* and *18S* sequences.

**Appendix S4:** ML phylogenetic tree inferred by IQ‐TREE from concatenated *COI + 18S* genes, using ‘full’ alignment dataset. alRT values below 70 and bootstrap values below 85 are not shown. Genus *Crenobia* is shaded in blue, cf. *Atrioplanaria* in orange and European *Phagocata* in green. Cave populations are marked by red letters. Where available, the number of pharynges is indicated to the right of species names. The presence or absence of eyes was analysed in cave populations only.
**Appendix S5:** ML phylogenetic tree inferred by IQ‐TREE from concatenated *COI + 18S* genes, using ‘gap50’ alignment dataset. alRT values below 70 and bootstrap values below 85 are not shown. Genus *Crenobia* is shaded in blue, cf. *Atrioplanaria* in orange, European *Phagocata* in green and the American *Phagocata*, with a misassigned sample from Spain, in yellow. Cave populations are marked by red letters. Where available, the number of pharynges is indicated to the right of species names. The presence or absence of eyes was plotted in cave populations only.
**Appendix S6:** ML phylogenetic tree inferred by IQ‐TREE from concatenated *COI + 18S* genes, using ‘trimmed’ alignment dataset. alRT values below 70 and bootstrap values below 85 are not shown. Genus *Crenobia* is shaded in blue and European *Phagocata* in green. The genus cf. *Atrioplanaria*, shaded in orange, is nested within the genus *Crenobia*. Cave populations are marked by red letters. Where available, the number of pharynges is indicated to the right of species names. The presence or absence of eyes was plotted in cave populations only.
**Appendix S7:** ML phylogenetic tree inferred by IQ‐TREE from *COI* gene, using ‘full’ alignment dataset. alRT values below 70 and bootstrap values below 85 are not shown. Genus *Crenobia* is shaded in blue, cf. *Atrioplanaria* in orange, European *Phagocata* with *P. torva* in green and the American *Phagocata*, with a *Phagocata* sp. sample from Spain, in yellow. Cave populations are marked by red letters. Where available, the number of pharynges is indicated to the right of species names. The presence or absence of eyes was plotted in cave populations only.
**Appendix S8:** ML phylogenetic tree inferred by IQ‐TREE from *COI* gene, using ‘gap50’ alignment dataset. alRT values below 70 and bootstrap values below 85 are not shown. Genus *Crenobia* is shaded in blue, cf. *Atrioplanaria* in orange, European *Phagocata* with *P. torva* in green and the American *Phagocata*, with a *Phagocata* sp. sample from Spain, in yellow. Cave populations are marked by red letters. Where available, the number of pharynges is indicated to the right of species names. The presence or absence of eyes was plotted in cave populations only.
**Appendix S9:** ML phylogenetic tree inferred by IQ‐TREE from *COI* gene, using ‘trimmed’ alignment dataset. alRT values below 70 and bootstrap values below 85 are not shown. Genus *Crenobia* is shaded in blue, cf. *Atrioplanaria* in orange, European *Phagocata* in green and the American *Phagocata* in yellow. Cave populations are marked by red letters. Where available, the number of pharynges is indicated to the right of species names. The presence or absence of eyes was plotted in cave populations only.
**Appendix S10:** ML phylogenetic tree inferred by IQ‐TREE from *18S* gene, using ‘full’ alignment dataset. alRT values below 70 and bootstrap values below 85 are not shown. Genus *Crenobia* is shaded in blue and the European *Phagocata* in green. The genus cf. *Atrioplanaria*, shaded in orange, is nested within the genus *Crenobia*. Cave populations are marked by red letters. Where available, the number of pharynges is indicated to the right of species names. The presence or absence of eyes was plotted in cave populations only.
**Appendix S11:** ML phylogenetic tree inferred by IQ‐TREE from *18S* gene, using ‘gap50’ alignment dataset. alRT values below 70 and bootstrap values below 85 are not shown. Branch lengths were transformed proportionally. Genus *Crenobia* is shaded in blue, and genus cf. *Atrioplanaria*, shaded in orange, is nested within *Crenobia*. Cave populations are marked by red letters. Where available, the number of pharynges is indicated to the right of species names. The presence or absence of eyes was plotted in cave populations only.
**Appendix S12:** ML phylogenetic tree inferred by IQ‐TREE from *18S* gene, using ‘trimmed’ alignment dataset. alRT values below 70 and bootstrap values below 85 are not shown. Branch lengths were transformed proportionally. Genus *Crenobia* is shaded in blue, and genus cf. *Atrioplanaria*, shaded in orange, is nested within *Crenobia*. Cave populations are marked by red letters. Where available, the number of pharynges is indicated to the right of species names. The presence or absence of eyes was plotted in cave populations only.
**Appendix S13:** BI phylogenetic tree inferred by MrBayes from *COI* gene, using ‘full’ alignment dataset. Posterior probabilities below 70 are not shown. Genus *Crenobia* is shaded in blue, cf. *Atrioplanaria* in orange, European *Phagocata* with *P. torva* in green and the American *Phagocata*, with a *Phagocata* sp. sample from Spain, in yellow. Cave populations are marked by red letters. Where available, the number of pharynges is indicated to the right of species names. The presence or absence of eyes was plotted in cave populations only.
**Appendix S14:** BI phylogenetic tree inferred by MrBayes from *18S* gene, using ‘full’ alignment dataset. Posterior probabilities below 70 are not shown. Genus *Crenobia* is shaded in blue, and the European *Phagocata* in green. Genus cf. *Atrioplanaria*, shaded in orange, is nested within *Crenobia*. Cave populations are marked by red letters. Where available, the number of pharynges is indicated to the right of species names. The presence or absence of eyes was plotted in cave populations only.
**Appendix S15:** Animals from cave1—Jama na Žutim kokom show variability in the presence of discernible eyes. (A) An animal with both eye spots. (B) An animal with one eye spot. (C) An animal with no discernible eyes. Full arrow heads point to visible eye spots. Empty arrow heads point to the area where eye spot is expected, but is not visible. Scale bar, 500 μm.

## Data Availability

All newly reported gene sequences have been submitted to the ENA public repository (bioProject accession PREJB112134).
