# Supplementary material for: Cave‐Dwelling Planariidae in Dinaric Karst Exhibit Differing Levels of Cave Trait Evolution
Source: Ecol Evol. 2026 Oct 28;16(11):e74488. doi: 10.1002/ece3.74488 (PMC13714693; doi:10.1002/ece3.74488)
Supplement: Supplementary file 1 [file ECE3-16-e74488-s004.pdf]

Appendix Figures and Legends S4 – S15

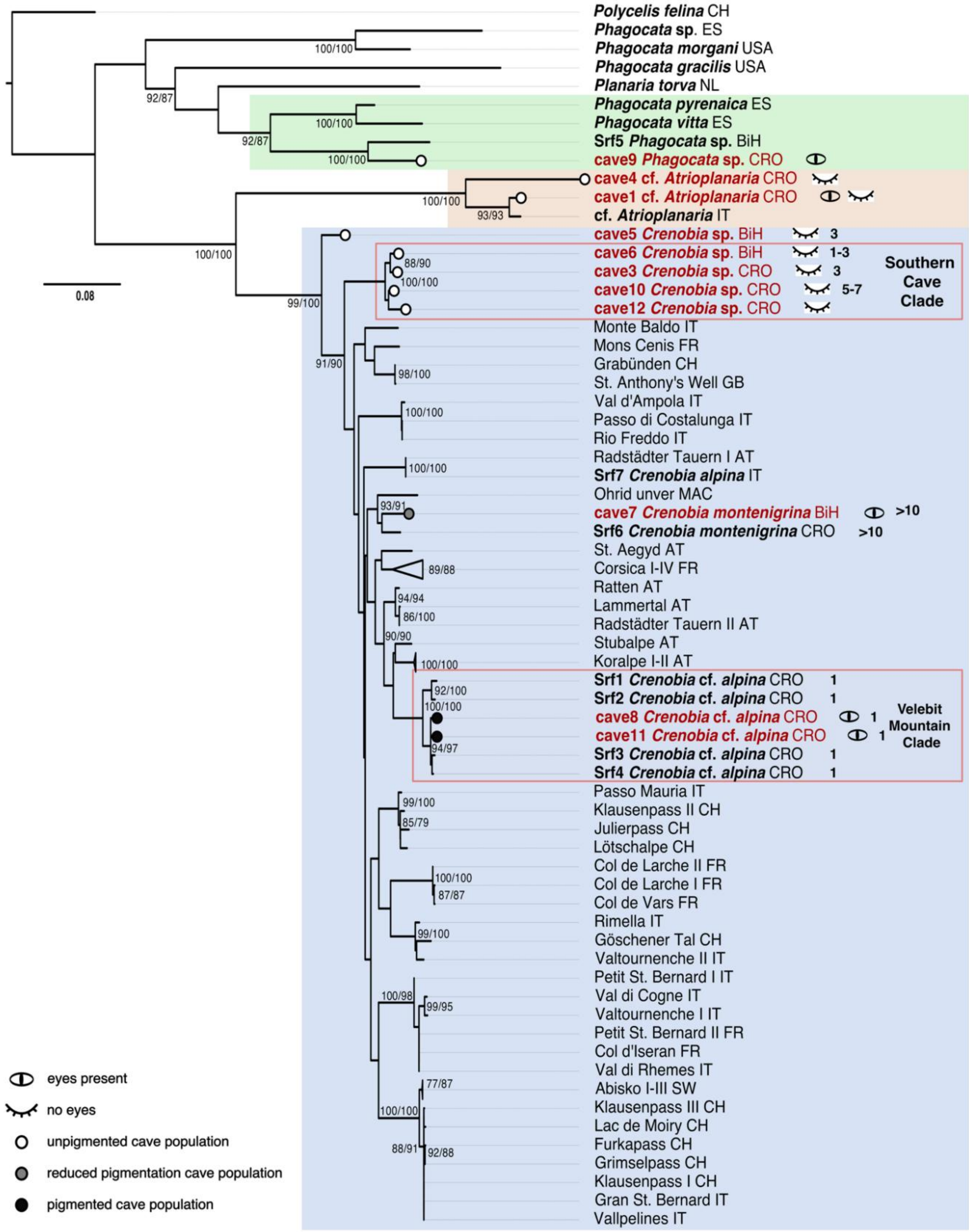

Appendix S4. ML phylogenetic tree inferred by IQ-TREE from concatenated *COI* + *18S* genes, using 'full' alignment dataset. aIRT values below 70 and bootstrap values below 85 are not shown. Genus *Crenobia* is shaded in blue, cf. *Atrioplanaria* in orange, and European *Phagocata* in green. Cave populations are marked by red letters. Where available, the number of pharynges is indicated to the right of species names. The presence or absence of eyes was analysed in cave populations only.

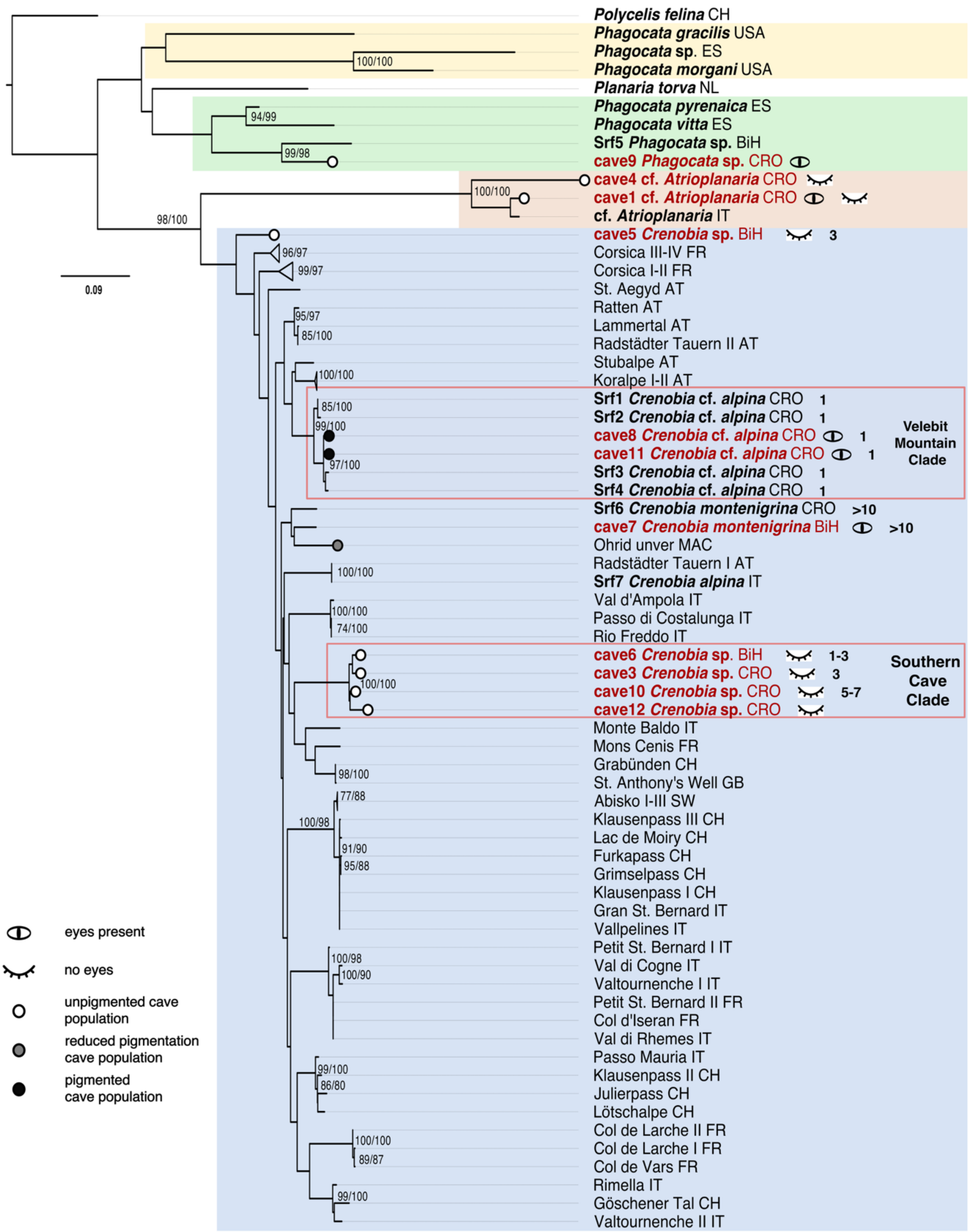

Appendix S5. ML phylogenetic tree inferred by IQ-TREE from concatenated *COI* + *18S* genes, using ‘gap50’ alignment dataset. aIRT values below 70 and bootstrap values below 85 are not shown. Genus *Crenobia* is shaded in blue, cf. *Atrioplanaria* in orange, European *Phagocata* in green, and the American *Phagocata,* with a misassigned sample from Spain, in yellow. Cave populations are marked by red letters. Where available, the number of pharynges is indicated to the right of species names. The presence or absence of eyes was plotted in cave populations only.

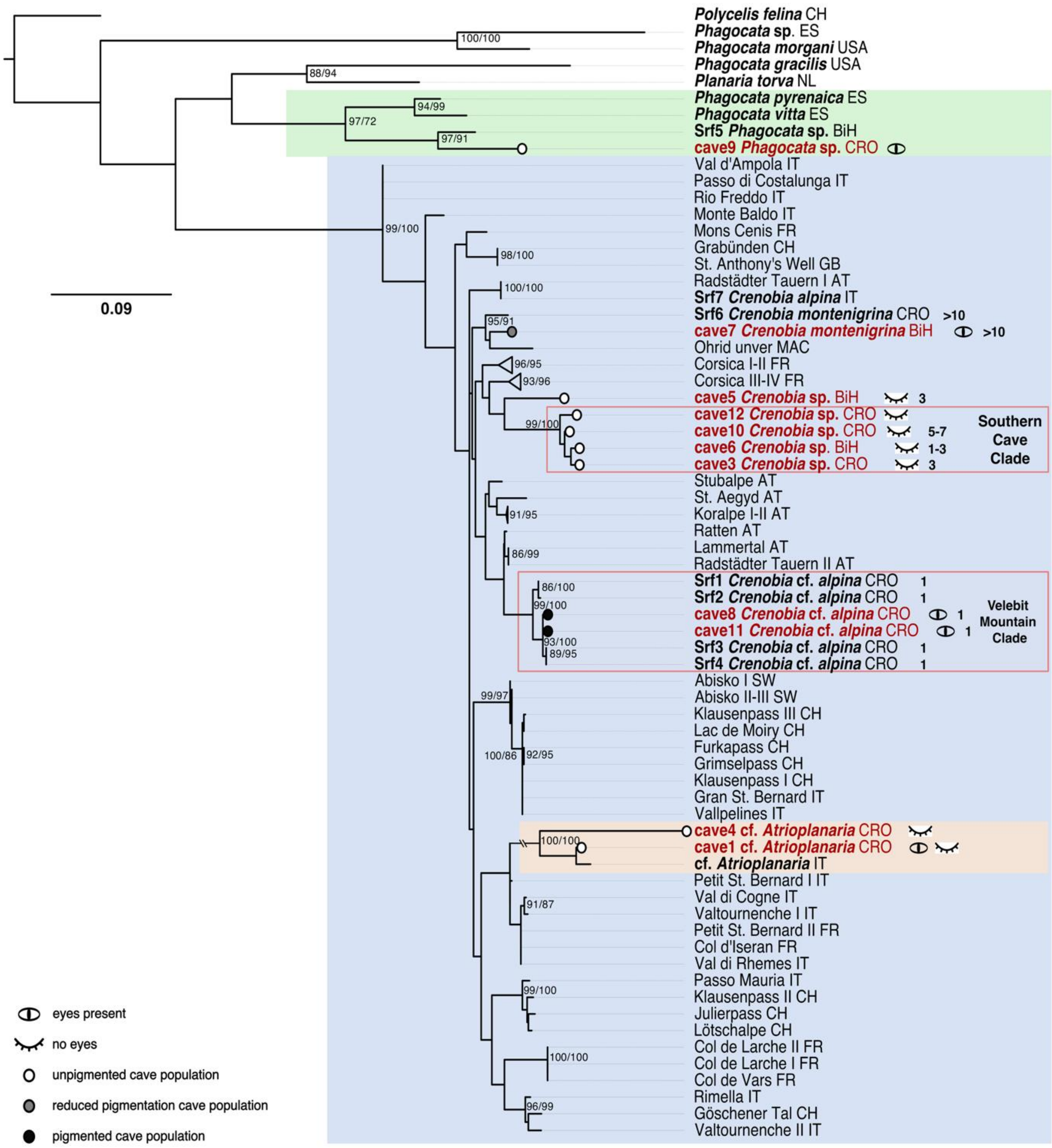

Appendix S6. ML phylogenetic tree inferred by IQ-TREE from concatenated *COI* + *18S* genes, using 'trimmed' alignment dataset. aIRT values below 70 and bootstrap values below 85 are not shown. Genus *Crenobia* is shaded in blue and European *Phagocata* in green. The genus cf. *Atrioplanaria*, shaded in orange, is nested within the genus *Crenobia.* Cave populations are marked by red letters. Where available, the number of pharynges is indicated to the right of species names. The presence or absence of eyes was plotted in cave populations only.

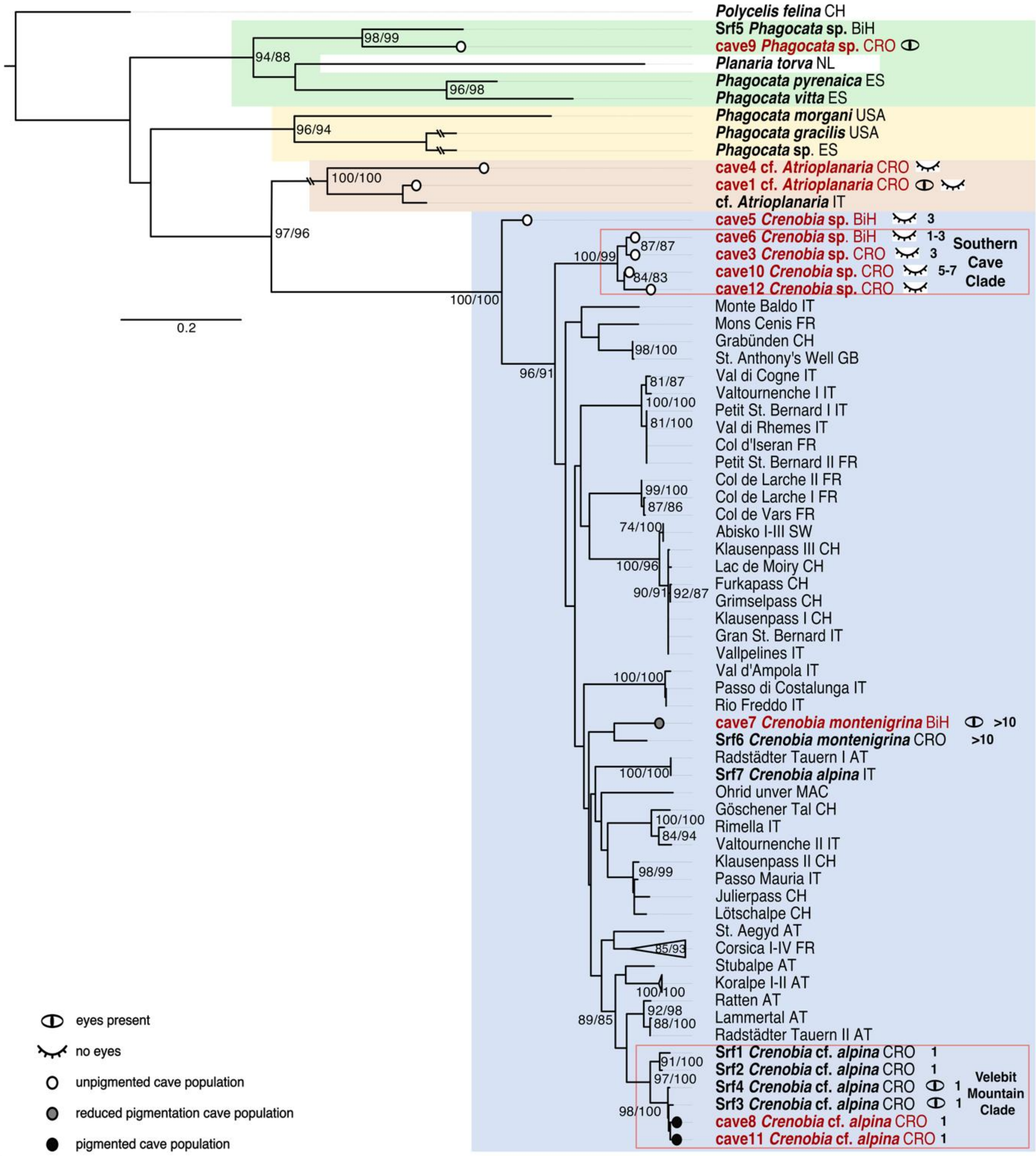

Appendix S7. ML phylogenetic tree inferred by IQ-TREE from *COI* gene, using 'full' alignment dataset. aIRT values below 70 and bootstrap values below 85 are not shown. Genus *Crenobia* is shaded in blue, cf. *Atrioplanaria* in orange, European *Phagocata* with *P. torva* in green, and the American *Phagocata,* with a *Phagocata* sp. sample from Spain, in yellow. Cave populations are marked by red letters. Where available, the number of pharynges is indicated to the right of species names. The presence or absence of eyes was plotted in cave populations only.

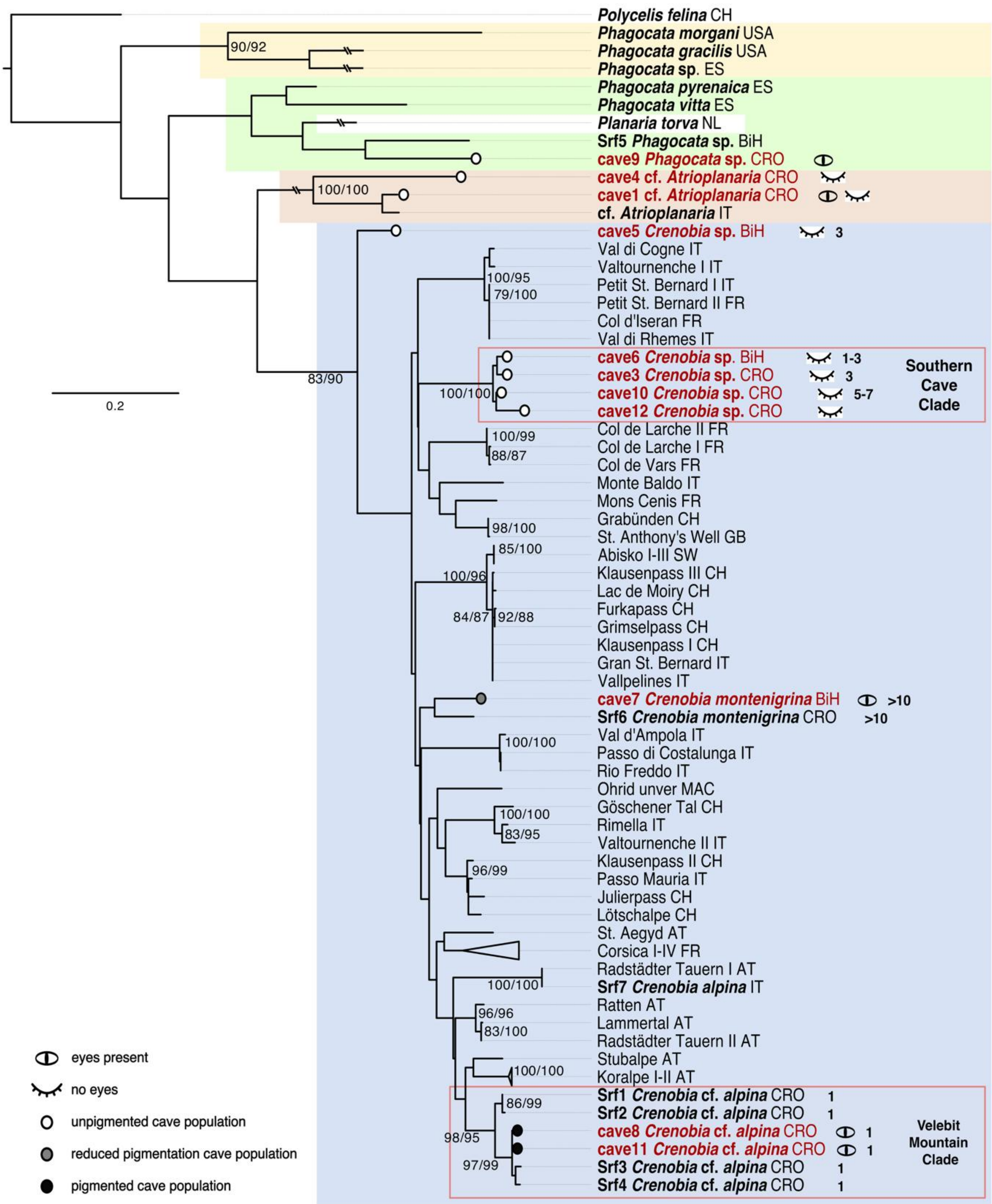

Appendix S8. ML phylogenetic tree inferred by IQ-TREE from *COI* gene, using ‘gap50’ alignment dataset. aIRT values below 70 and bootstrap values below 85 are not shown. Genus *Crenobia* is shaded in blue, cf. *Atrioplanaria* in orange, European *Phagocata* with *P. torva* in green, and the American *Phagocata,* with a *Phagocata* sp. sample from Spain, in yellow. Cave populations are marked by red letters. Where available, the number of pharynges is indicated to the right of species names. The presence or absence of eyes was plotted in cave populations only.

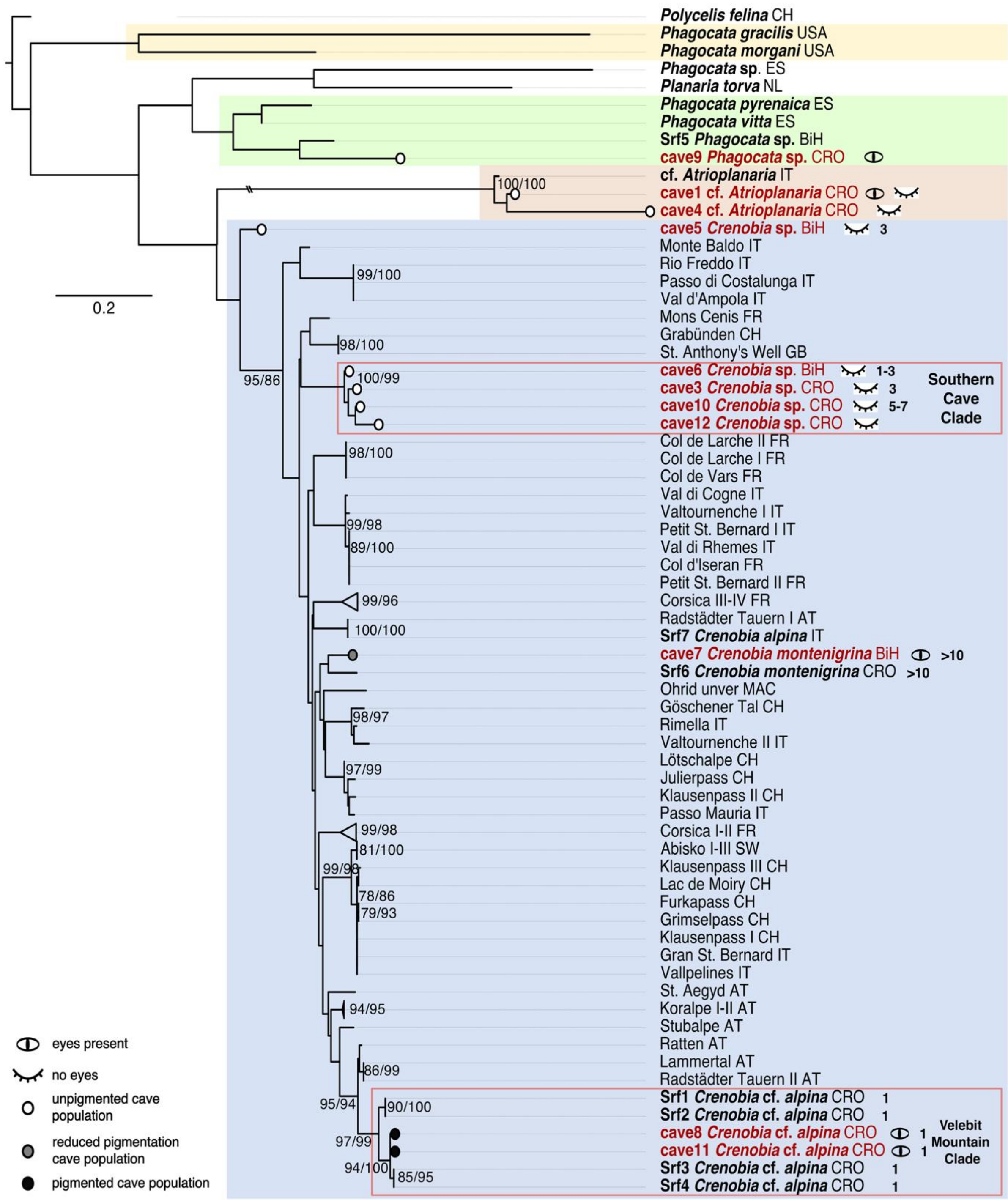

Appendix S9. ML phylogenetic tree inferred by IQ-TREE from *COI* gene, using 'trimmed' alignment dataset. aIRT values below 70 and bootstrap values below 85 are not shown. Genus *Crenobia* is shaded in blue, cf. *Atrioplanaria* in orange, European *Phagocata* in green, and the American *Phagocata* in yellow. Cave populations are marked by red letters. Where available, the number of pharynges is indicated to the right of species names. The presence or absence of eyes was plotted in cave populations only.

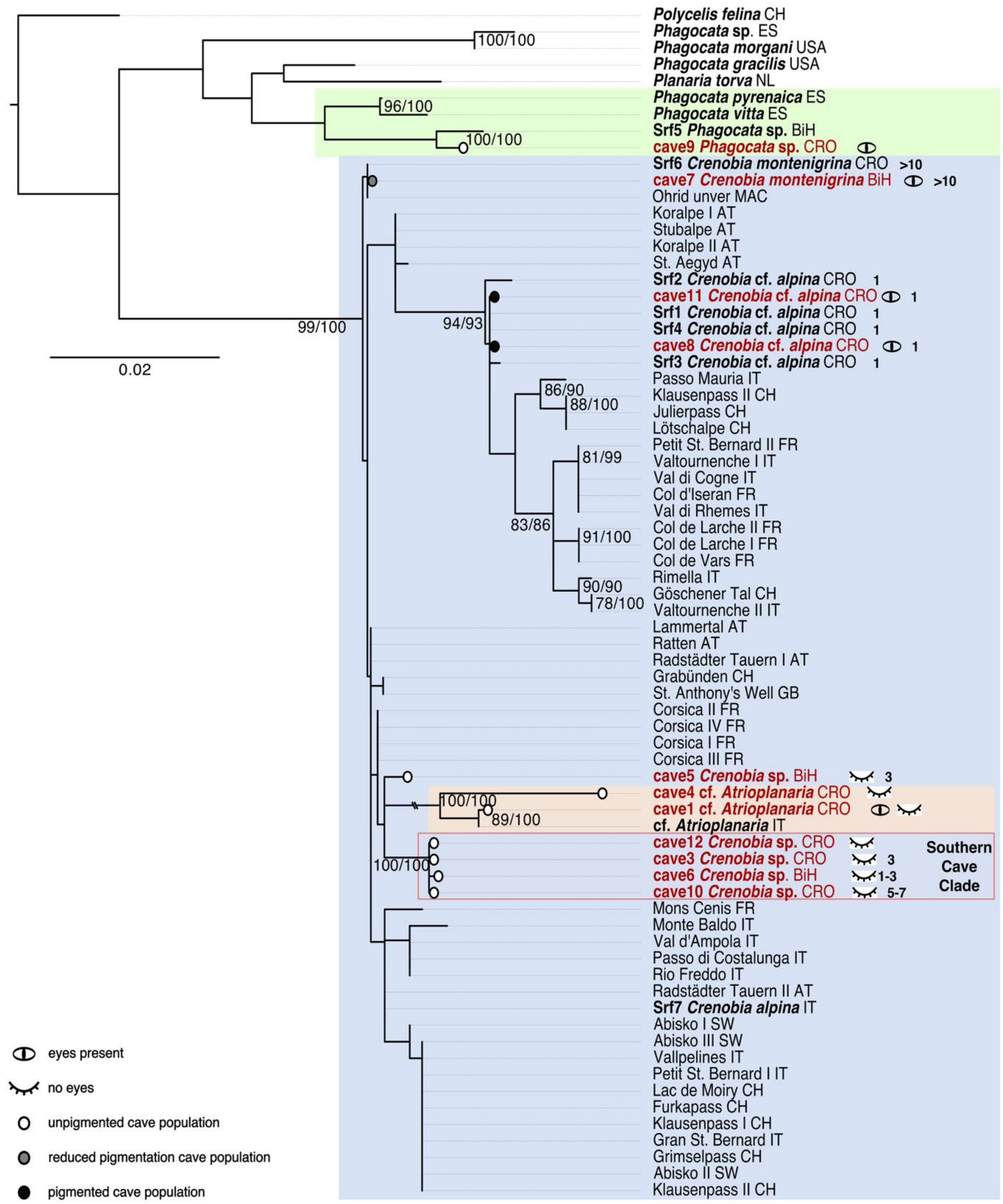

Appendix S10 ML phylogenetic tree inferred by IQ-TREE from *18S* gene, using 'full' alignment dataset. aIRT values below 70 and bootstrap values below 85 are not shown. Genus *Crenobia* is shaded in blue and the European *Phagocata* in green. The genus cf. *Atrioplanaria*, shaded in orange, is nested within the genus *Crenobia.* Cave populations are marked by red letters. Where available, the number of pharynges is indicated to the right of species names. The presence or absence of eyes was plotted in cave populations only.

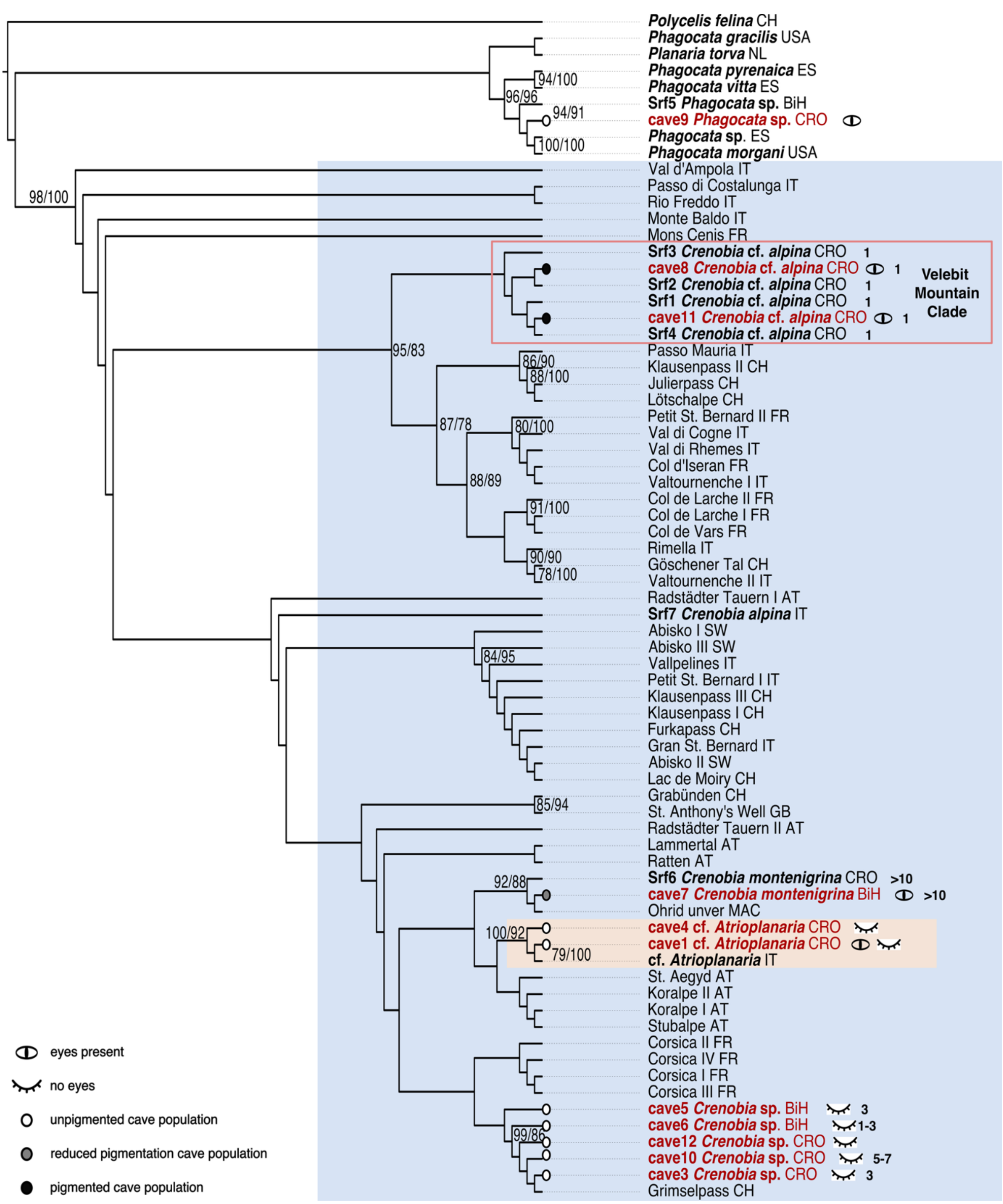

Appendix S11. ML phylogenetic tree inferred by IQ-TREE from *18S* gene, using 'gap50' alignment dataset. aIRT values below 70 and bootstrap values below 85 are not shown. Branch lengths were transformed proportionally. Genus *Crenobia* is shaded in blue, and genus cf. *Atrioplanaria*, shaded in orange, is nested within *Crenobia.* Cave populations are marked by red letters. Where available, the number of pharynges is indicated to the right of species names. The presence or absence of eyes was plotted in cave populations only.

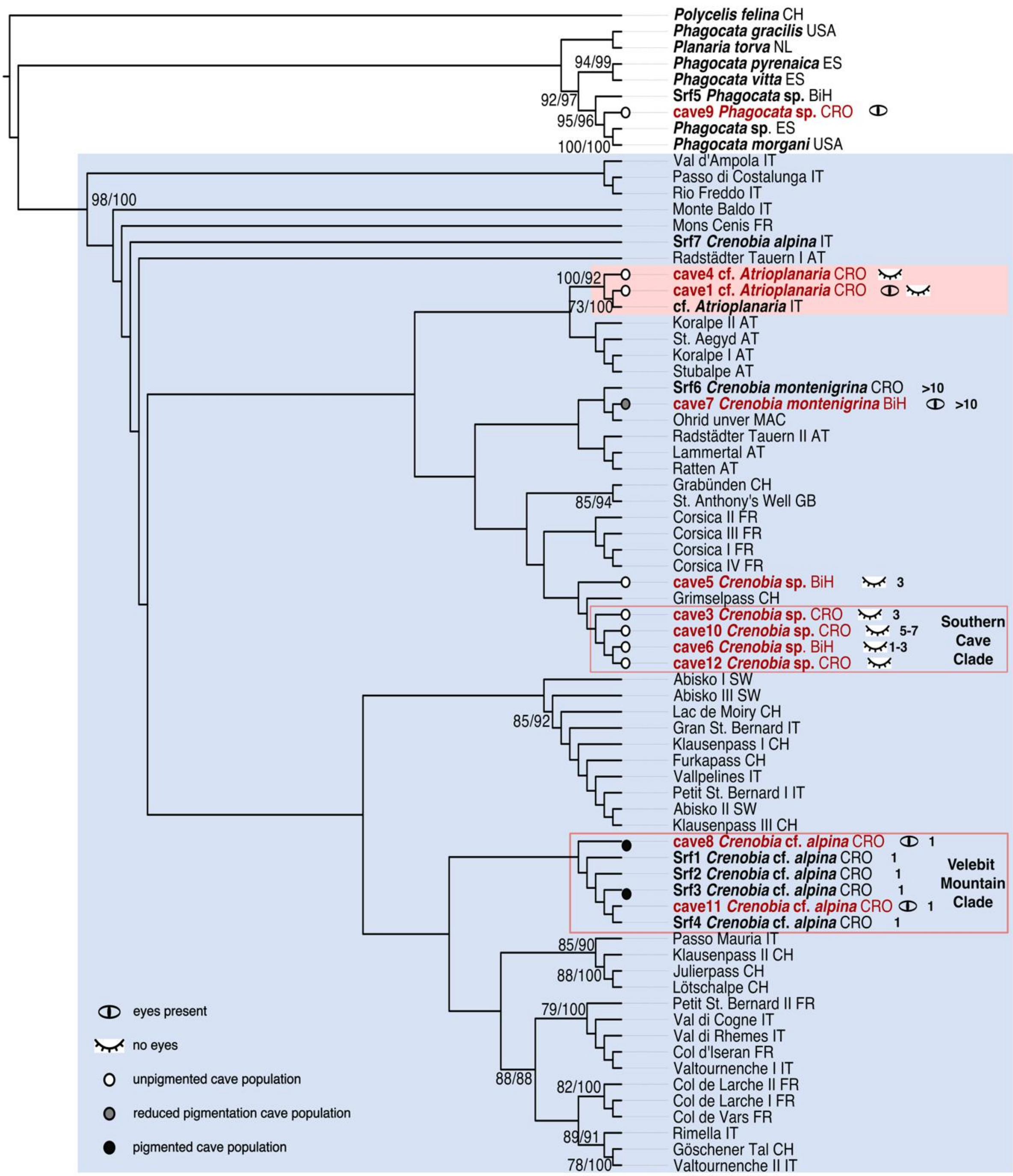

Appendix S12. ML phylogenetic tree inferred by IQ-TREE from *18S* gene, using 'trimmed' alignment dataset. alRT values below 70 and bootstrap values below 85 are not shown. Branch lengths were transformed proportionally. Genus *Crenobia* is shaded in blue, and genus cf. *Atrioplanaria*, shaded in orange, is nested within *Crenobia.* Cave populations are marked by red letters. Where available, the number of pharynges is indicated to the right of species names. The presence or absence of eyes was plotted in cave populations only.

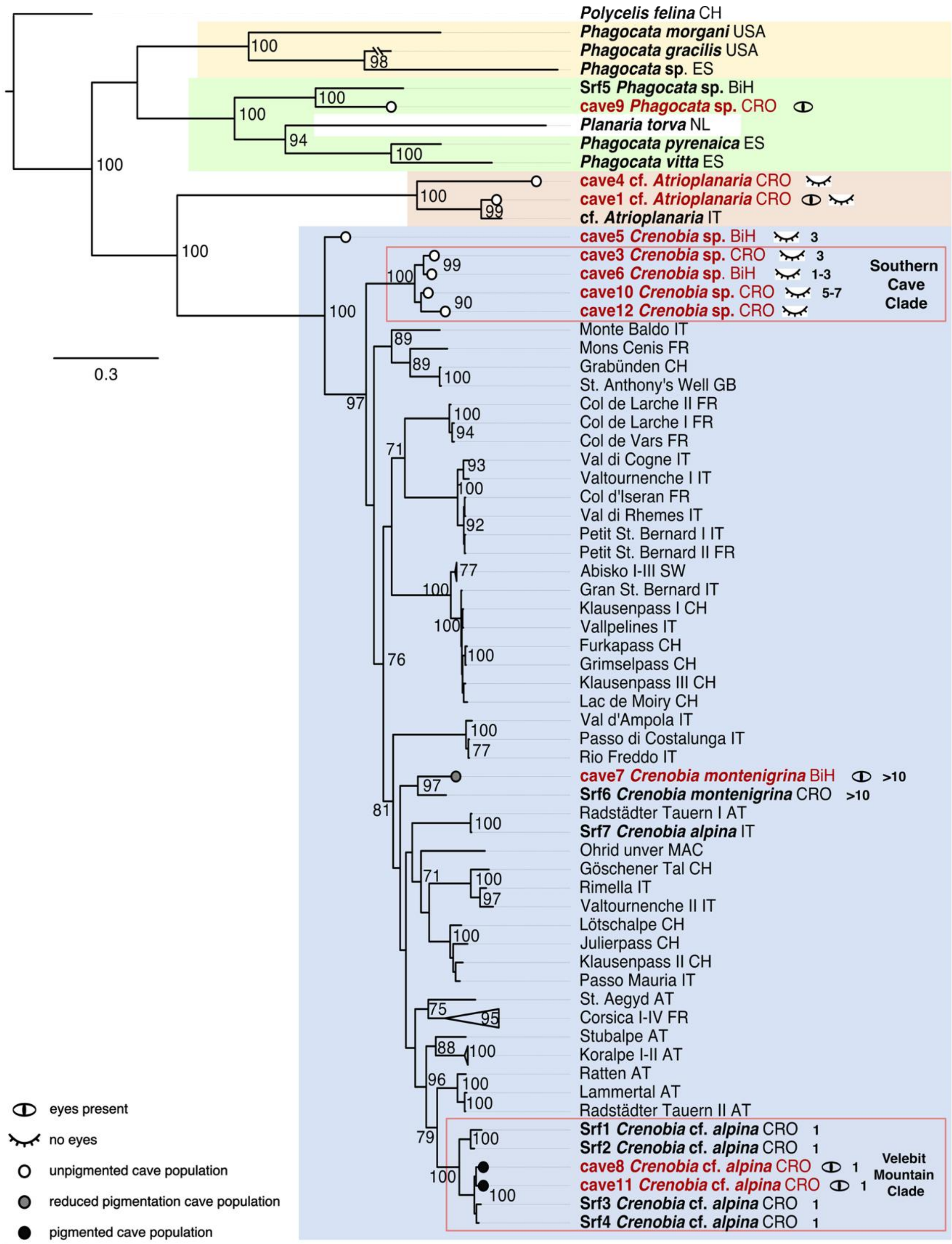

Appendix S13. BI phylogenetic tree inferred by MrBayes from *COI* gene, using 'full' alignment dataset. Posterior probabilities below 70 are not shown. Genus *Crenobia* is shaded in blue, cf. *Atrioplanaria* in orange, European *Phagocata* with *P. torva* in green, and the American *Phagocata,* with a *Phagocata* sp. sample from Spain, in yellow. Cave populations are marked by red letters. Where available, the number of pharynges is indicated to the right of species names. The presence or absence of eyes was plotted in cave populations only.

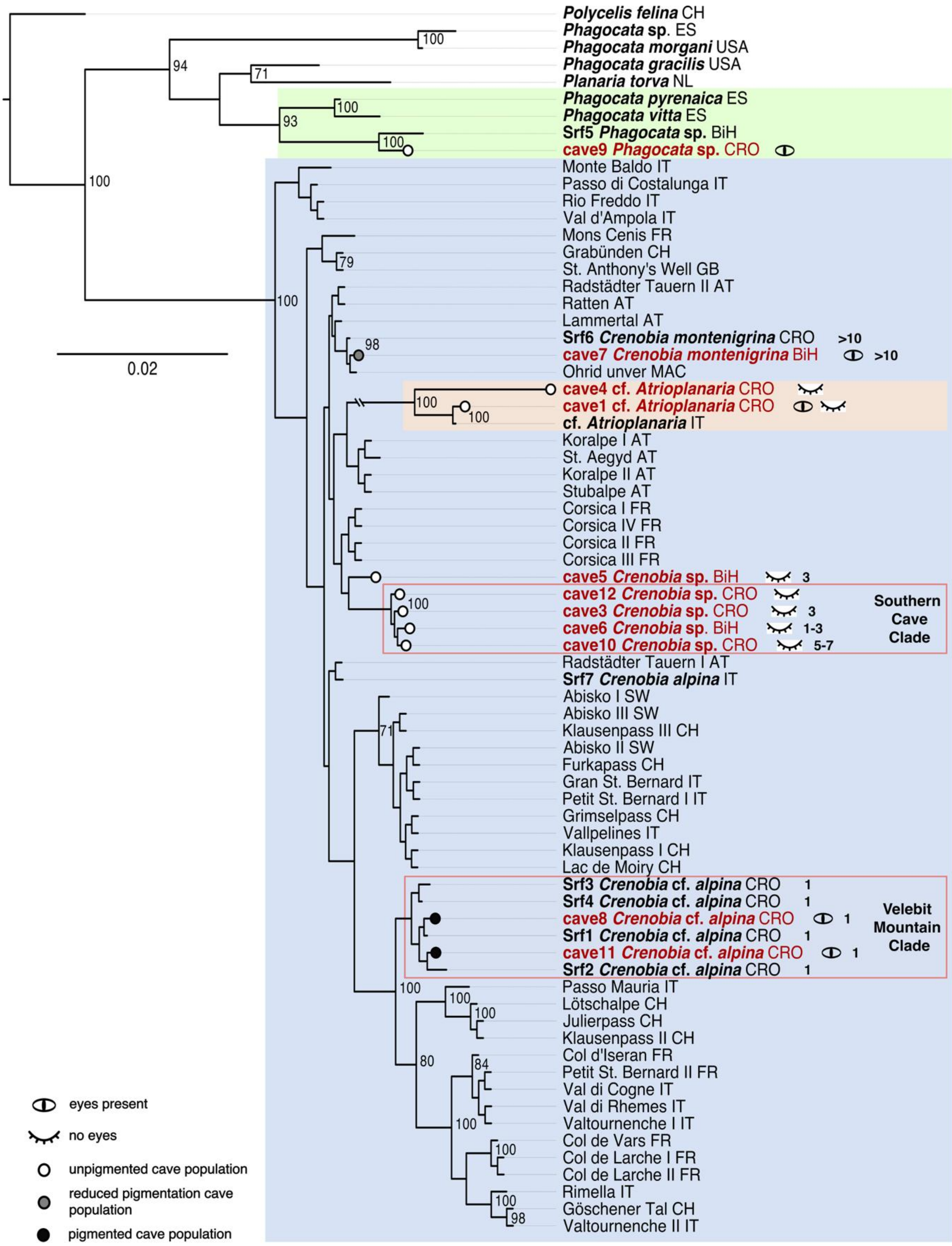

Appendix S14. BI phylogenetic tree inferred by MrBayes from *18S* gene, using 'full' alignment dataset. Posterior probabilities below 70 are not shown. Genus *Crenobia* is shaded in blue, and the European *Phagocata* in green. Genus cf. *Atrioplanaria*, shaded in orange, is nested within *Crenobia.* Cave populations are marked by red letters. Where available, the number of pharynges is indicated to the right of species names. The presence or absence of eyes was plotted in cave populations only.

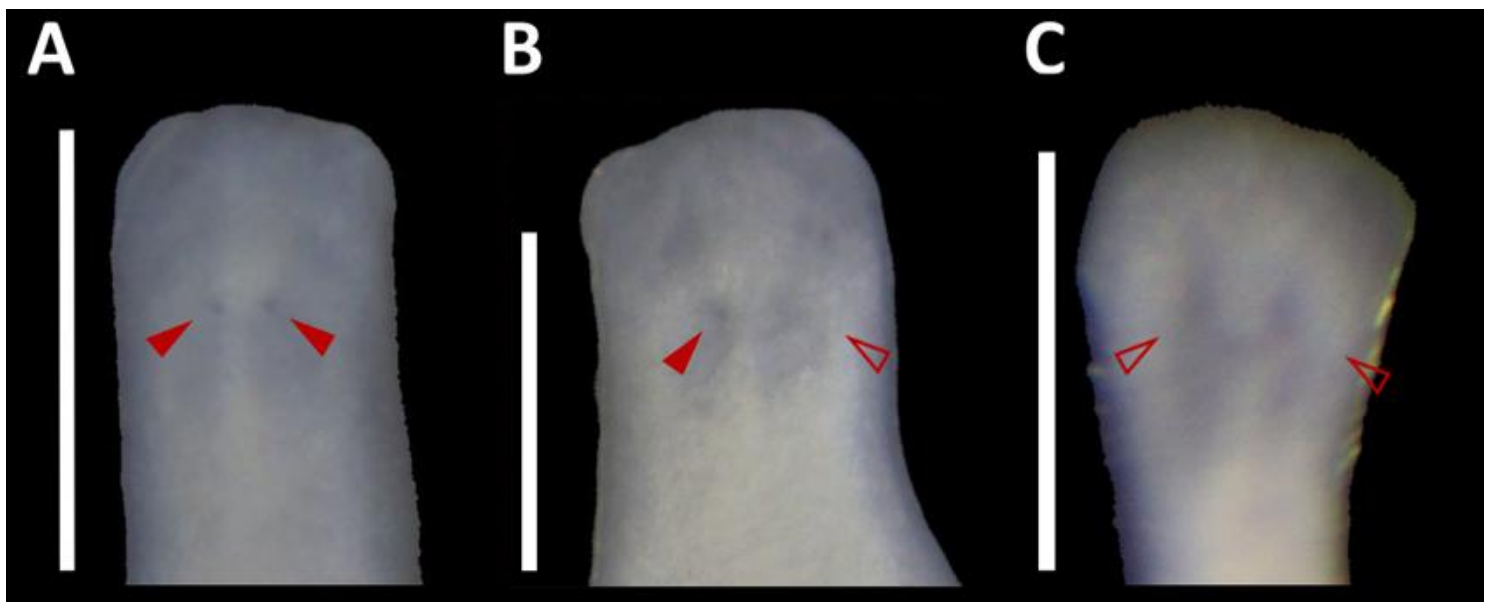

Appendix S15. Animals from cave1 - Jama na Žutim kokom show variability in the presence of discernible eyes. **A** An animal with both eye spots. **B** An animal with one eye spot. **C** An animal with no discernible eyes. Full arrow heads point to visible eye spots. Empty arrow heads point to the area where eye spot is expected, but is not visible. Scale bar, 500 µm.
